# Supplementary material for: Proximity-induced supercurrent through topological insulator based nanowires for quantum computation studies
Source: Sci Rep. 2018 Nov 22;8:17237. doi: 10.1038/s41598-018-35424-1 (PMC6250704; doi:10.1038/s41598-018-35424-1)
Supplement: Supplementary file 1 — Supplementary Information [file 41598_2018_35424_MOESM1_ESM.pdf]

Supplementary Materials

Proximity-induced supercurrent through topological insulator based nanowires for quantum computation studies

Biplab Bhattacharyya,[1, 2] V. P. S. Awana,[1, 2] T. D. Senguttuvan,[1, 2] V. N. Ojha,[1, 2] Sudhir Husale[1, 2]*

[1] Academy of Scientific and Innovative Research (AcSIR), National Physical Laboratory, Council of Scientific and Industrial Research, Dr. K. S Krishnan Road, New Delhi-110012, India.

[2] National Physical Laboratory, Council of Scientific and Industrial Research, Dr. K. S Krishnan Road, New Delhi-110012, India.

*E-mail: husalesc@nplindia.org

Contents:

1. Higher magnification FESEM images
2. HRTEM characterization of W-Bi_2Se_3 interface
3. Absence of FIB-induced Halo effect during electrode deposition
4. Magnetoresistance (MR) at 10K for device N5

1. Higher magnification FESEM images

Figure S1 shows the higher magnification FESEM images of FIB deposited W electrodes on Bi_2Se_3 nanowires. Clean Bi_2Se_3 channel lengths can be clearly observed with little contamination or diffusion of W limited very close to the edges of the electrodes within 40 to 50 nm lengths from electrode edge. This is suggestive of the fact that with controlled FIB deposition parameters, electrodes can be deposited at < 500 nm gap lengths to perform such supercurrent studies. We have used optimized and controlled electrode deposition parameters and taken careful steps to keep the Ga ion contamination to minimum during fabrication process. Sharp Bi_2Se_3 nanowire sidewalls with missing granular W nano-islands on the substrate surface (in dark black with no white nano-clusters) are clearly visible in these FESEM images. Thus, indicating that most of the TI junction length is free from W contamination.

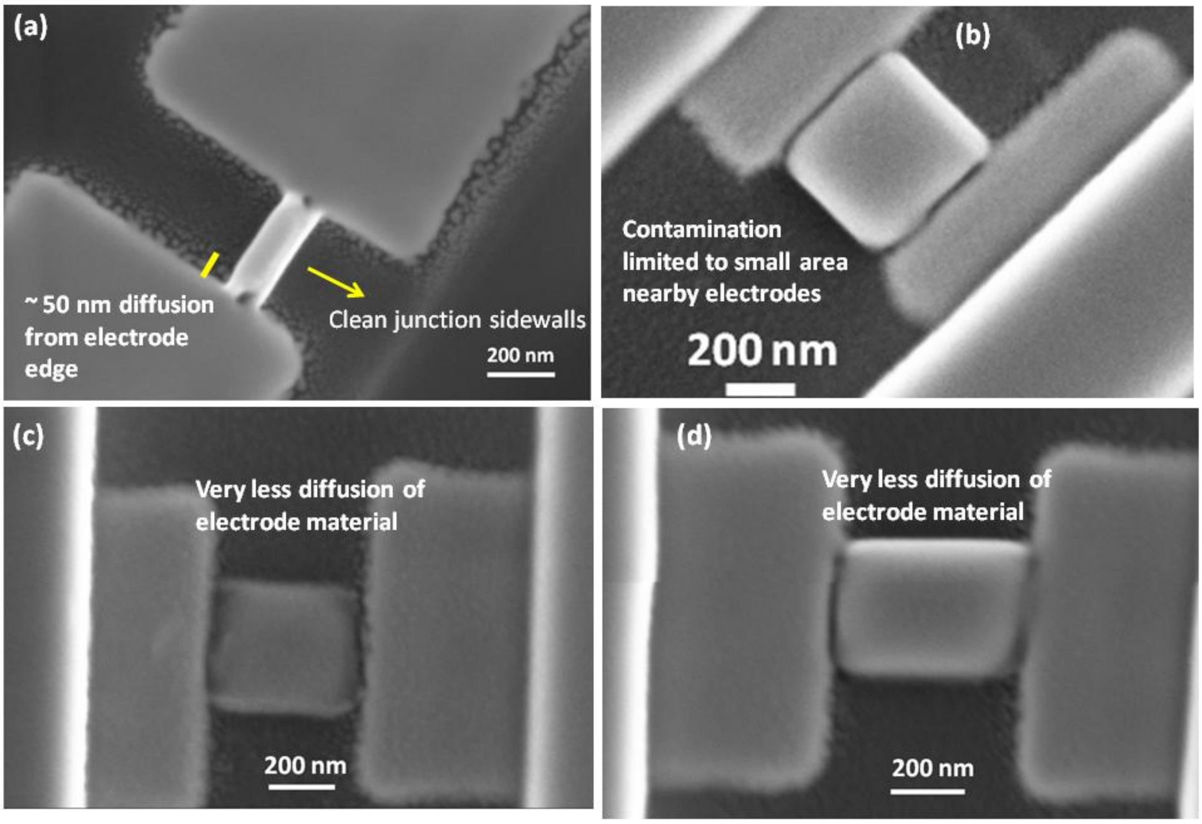

Fig. S1. Higher magnification FESEM images of Bi_2Se_3 nanowire devices. (A) Device N5 used in manuscript. **(A-D)** Sharp nanowire junction sidewalls are clearly visible in all the fabricated devices. Tiny granular nanoislands are present only at the interface within ~ 50 nm or less, keeping most of the junction length clean.

2. HRTEM characterization of W-Bi_2Se_3 interface

To carry out the HRTEM characterization for interface study of W-Bi_2Se_3 heterostructure, we deposited flakes of Bi_2Se_3 on TEM grid and used FIB to deposited W contact on the flake. Here, we show the FESEM images (Fig. S2) of the deposited Bi_2Se_3 flake and W contact on TEM grid. The high magnification FESEM images in Fig. S2 (B and C) show clear interfaces with no granular islands. The W electrode has very sharp edges and sidewalls, with very restricted diffusion and contamination in the electrodes surroundings. This is a result of substrate dependent deposition optimized conditions.

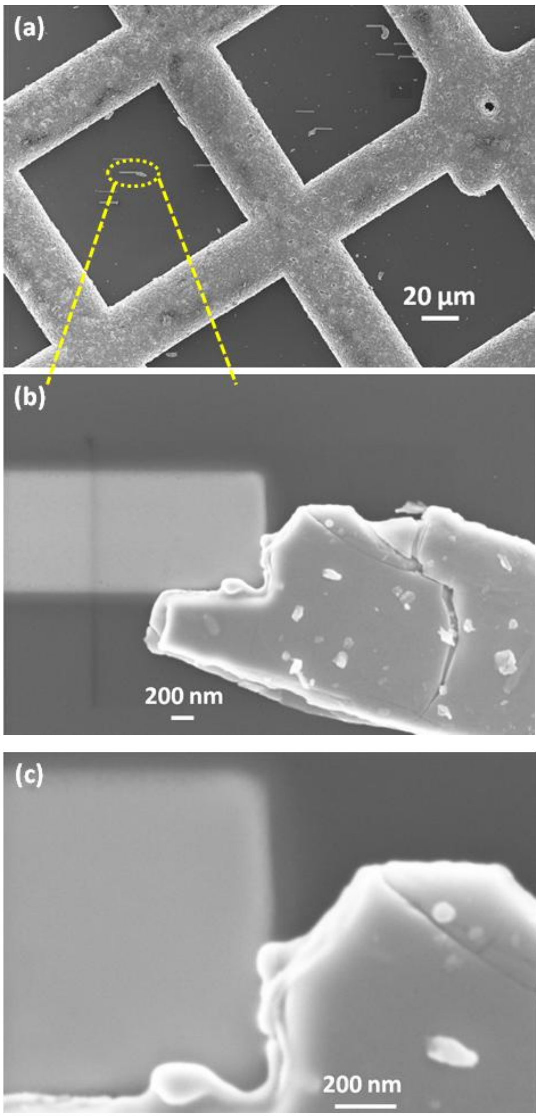

Fig. S2. FESEM images of Bi_2Se_3 flake and FIB deposited W contact on TEM grid. (A) Bi_2Se_3 flake (random shaped) and FIB deposited W electrode (straight horizontal bars) deposited on a TEM grid. **(B)** Higher magnification image of the encircled region in (A) showing the flake and electrode with more clarity. **(C)** Further higher magnification of the interface region between W and Bi_2Se_3. Very less diffusion or contamination can be observed on the flake or on the surroundings of the electrode. W electrode has sharp edges and sidewalls.

Figure S3 depicts the TEM images of the FIB deposited W electrodes on TEM grid. Figure S2 (a) shows the W electrodes with very good deposition parameters. The magnified electrode and edge region in Fig. S2 (b) shows the small area diffusion of W material in the surroundings of the electrode. The contamination is limited to roughly around 50 nm from the electrode edge.

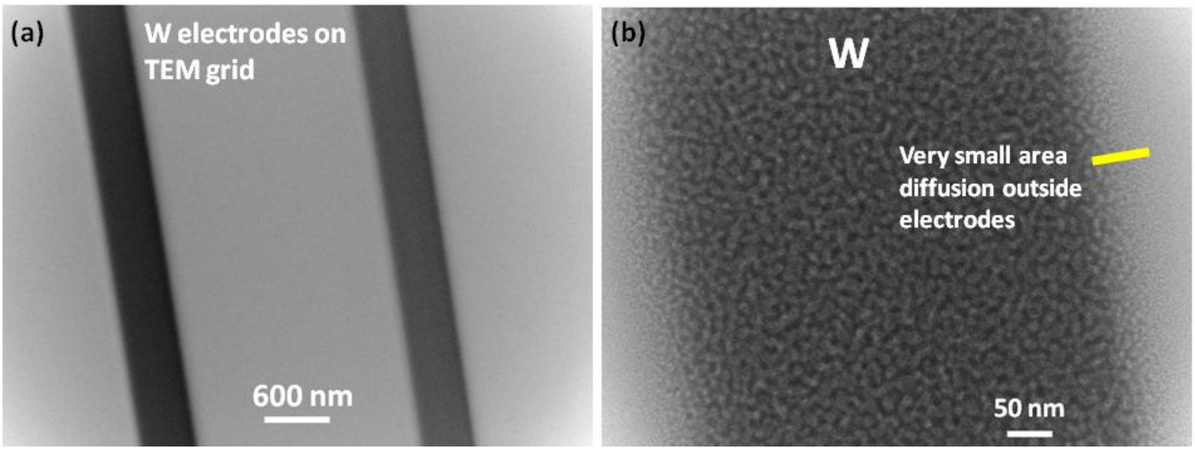

Fig. S3. TEM images of the W electrodes on TEM grid. (A) W electrodes with very sharp edges and sidewalls. **(B)** Very less diffusion of W material in the neighborhood of electrode. The contamination is limited within ~50 nm from electrode edge.

Figure S4 depicts the HRTEM images of the W electrode in contact with Bi_2Se_3 flake deposited on TEM grid. We can clearly see in the magnified view of the interface that crystalline Bi_2Se_3 planes start to emerge after some distance from the electrode edge and this distance was again found to be on an average ~50 to 60 nm from the electrode edge. In the area within 50 nm from the electrode edge, no clear crystalline planes were observed, which reflects the fact that the material may have become amorphous in that region and perfect Bi_2Se_3 crystal structure may have got distorted in that region. But, overall we find that the contamination is limited to the area closer to the electrode edges.

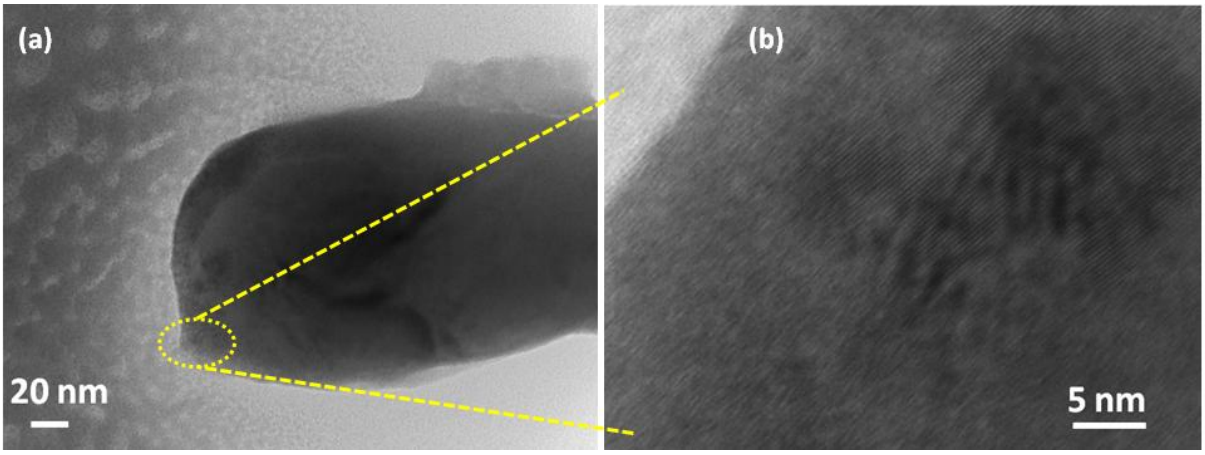

Fig. S4. HRTEM characterization of the W- Bi2Se3 interface. (a) Interface of W-Bi_2Se_3. **(B)** Higher magnification image of the region shown by yellow dashed circle in (A). Clear crystalline planes can be seen to emerge in Bi_2Se_3 flake after some distance (~50 to 60 nm) from electrode edge.

3. Absence of FIB-induced Halo effect during electrode deposition

The issue of W electrode deposition induced contamination has been taken into consideration in the control experiments. We have deposited only short channel electrodes (with no Bi_2Se_3 nanowire) on SiO_2/Si substrate (Fig. S5). But, we have found that such nano-gaps are highly insulating in nature, with resistance greater than 50 MΩ. Thus we have concluded that, in case of high quality of nanodevices (very less nano-grains), deposited impurities are insulating and cannot affect the transport properties of TIs. Hence, we believe that the Halo effect is not an issue in our devices, which again strengthens our claim that the observed exciting and unconventional superconductivity results are due to the properties of Bi_2Se_3 nanowire junction.

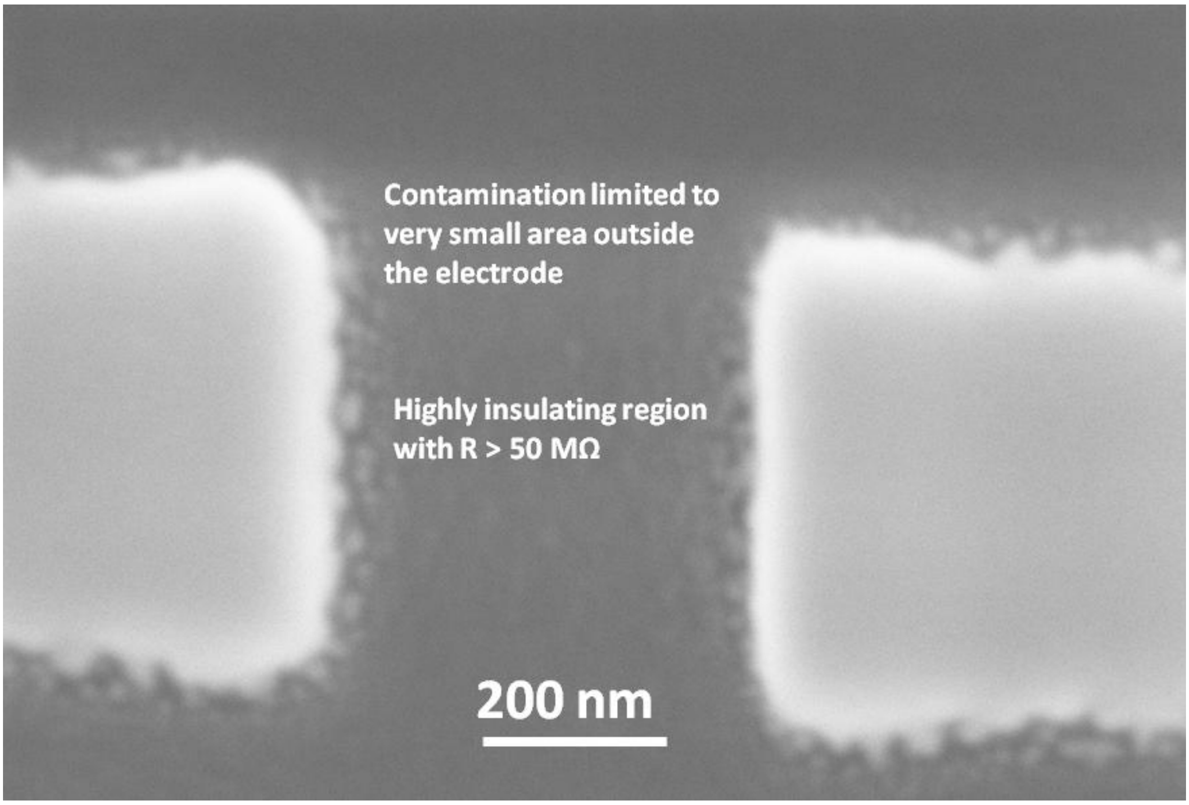

Fig. S5. Control experiments to confirm the absence of halo effect. High magnification FESEM image of FIB deposited W electrodes to measure the extent of halo effect through electrical resistance measurement between the nano-gaps. The measured electrical resistance is > 50 MΩ in the nano-gaps.

4. Magnetoresistance (MR) at 10K for device N5

We have analysed the MR data for device N5 in Fig. 1B of the main manuscript based on the theory of TIs and their behaviour in perpendicular B-field. Figure S6 shows the comparison in MR (R-R (0 T)) at 2 K and 10 K, where the R (0 T) at 2 K and 10 K is 0 Ω and 566.62 Ω, respectively.

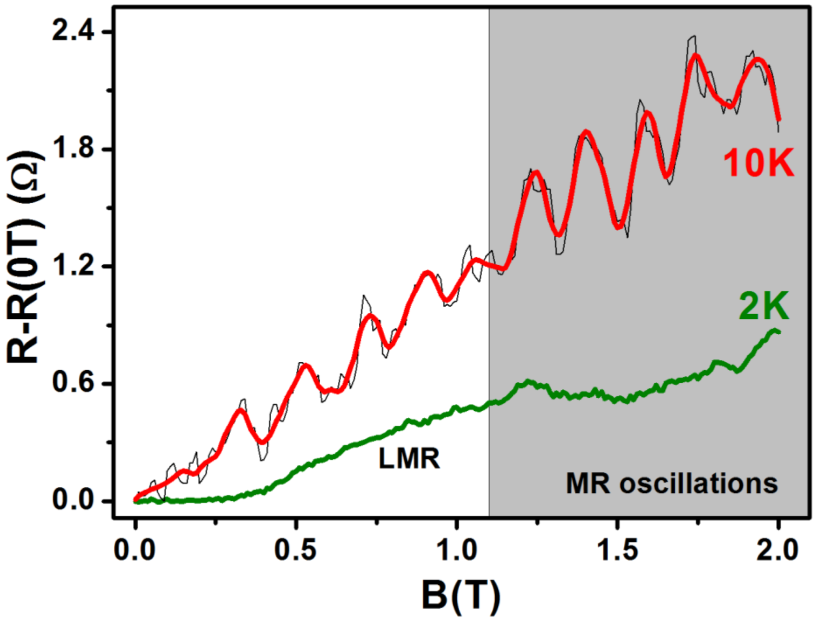

Fig. S6. MR at 2 K and 10 K for device N5. Since the nanowire becomes superconducting at 2 K, a zero resistance is observed at lower B-fields with LMR and MR oscillations emerging at higher B-fields. For 10 K, clear MR oscillations under perpendicular B-field with increasing oscillation amplitude with B-field can be observed.

The MR at 10 K has been smoothed (red colour) only to clearly visualize the features in the signal. A positive MR with increasing oscillation amplitude is visible at 10 K (> T_c), where sample N5 is completely in the normal state with zero field resistance around 567 Ω. The behaviour at 10 K corresponds to the SdH effect of very low cyclotronic mass fermions ($\omega_c = eB/m_c$) as under perpendicular B-field electrons perform cyclotronic motion that leads to Landau quantization of these orbits with a frequency spacing of ω_c. The dominance of these oscillations at significantly lower B-fields (~ 2 T) suggests the possibility of very low mass fermions present on the surface of TIs. As we shift to a temperature below T_c, the MR at 2K shows a completely superconducting state with zero resistance at low B-fields, which is possibly due to the proximity-induced superconductivity in TI. With increasing B-field, first a linear MR (LMR) is observed and then oscillatory features are observed at fields above 1T. Now, we believe that these features (when superconductivity is lost due to B-field) may be arising from the TSS. Thus, we argue in the manuscript that a topological superconducting (TSC) phase has been realized at 2K.
